# Hemothorax From an Anomalous Bronchial Artery Bleed in an Infected Intralobar Pulmonary Sequestration

**DOI:** 10.7759/cureus.28656

**Published:** 2022-08-31

**Authors:** Mohammad Anwar

**Affiliations:** 1 General Medicine, Hampshire Hospitals NHS Foundation Trust, Basingstoke, GBR

**Keywords:** senior løken syndrome, pulmonary lobectomy, pseudomonas infections, bronchial artery embolization (bae), hemothorax, bronchial artery, extralobar pulmonary sequestration, intralobar pulmonary sequestration, pulmonary sequestration

## Abstract

Pulmonary sequestration is a rare congenital malformation. It represents 0.15-6.4% of all congenital pulmonary malformations. It is characterized by non-functional, dysplastic mass of lung tissue that is not in communication with the normal tracheobronchial tree and is associated with a systemic arterial supply. We report a young gentleman in his mid-thirties who presented with community-acquired pneumonia from an infected intralobar pulmonary sequestration which subsequently developed a hemothorax from an anomalous bronchial artery bleed.

## Introduction

Pulmonary sequestrations can be sub-divided on anatomical basis as either intra-lobar sequestration (ILS) in which the sequestration lies within the native lung’s visceral pleural lining or extra-lobar sequestration (ELS), which has its own pleural investment. ILS is more common (75% of cases) and presents in early adulthood usually with recurrent pneumonia. Diagnosis is usually confirmed with computed tomography/magnetic resonance angiogram. Treatment is usually embolization of the anomalous artery with surgical removal of the sequestration.

## Case presentation

A middle-aged gentleman in his thirties presented to the emergency department (ED) with shortness of breath on exertion with a productive cough for four days, three episodes of vomiting in three days and one-day history of right-sided chest/epigastric/right flank pain which initially radiated to his right shoulder. The pain was continuous and worse on coughing and taking a deep breath in. He also had intermittent fever up to 38.8C. He saw his general practitioner (GP) one day before admission. The GP had prescribed oral amoxicillin but the patient’s symptoms did not improve so he presented to the emergency department. He was given 1g Amoxicillin IV, 500mg PO clarithromycin, 1g Paracetamol IV and 4mg IV Ondansetron in ED, with 1 L IV fluids.

His previous medical history included: hypertension, IgA nephropathy, chronic kidney disease stage 3, right kidney transplant, Senior Loken syndrome, supra-ventricular tachycardia, 1st-degree hemorrhoids, chromosome 2 deletion also chromosome 4 deletion including ANK2 gene, sectoral retinitis pigmentosa, Right Ramsay Hunt syndrome and Bell's palsy.

His regular medications included Prednisolone 5mg OD, Tacrolimus 2mg OD and Mycophenolate 1g BD. His observations were within normal limits except tachycardia of eighty-six (86) bpm. On examination, he had decreased air entry and crepitations on the right side of the chest. The rest of the general physical examination including heart sounds, abdomen and legs/calves was unremarkable.

**Figure 1:**
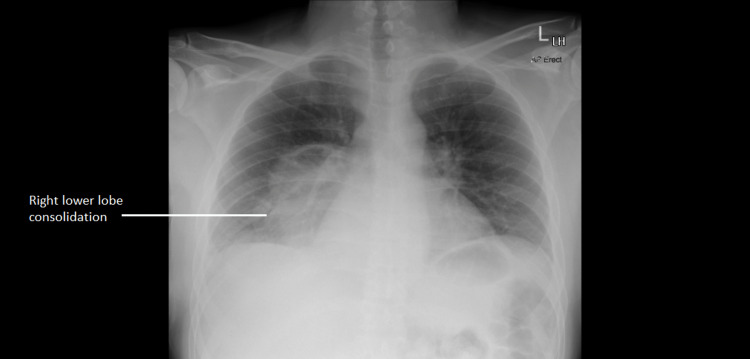
Chest X-ray showing right lower lobe consolidation

His blood tests (see Table 1 below) showed a raised C-reactive protein (CRP) (344 mg/L), procalcitonin was also raised (20.59 ug/L), raised D-dimer (1.47 ugFEU/ml), with reduced glomerular filtration rate (GFR) (35 ml/min) (patient's baseline was around 58 ml/min), and raised creatinine (201 umol/L) (patient's baseline was around 130 umol/L). His platelet count, WBC, haemoglobin, and liver function tests were unremarkable except for a raised bilirubin (32 umol/L). His chest X-ray showed right lower lobe consolidation (Figure 1).

**Table 1:** Blood test results

Blood Test	Result	Reference Value
C-reactive protein	344 mg/L	0-5 mg/L
Procalcitonin	20.59 ug/L	0-0.25 ug/L
Hemoglobin	164 g/L	130-180 g/L
White blood cell count	10.0 x 10^9^/L	4-11 x 10^9^/L
Platelet count	200 x 10^9^/L	150-500 x 10^9^/L
Neutrophil count	8.5 x 10^9^/L	1.5-8 x 10^9^/L
GFR (glomerular filtration rate)	35 ml/min	60-150 ml/min
Creatinine	201 umol/L	59-104 umol/L
Bilirubin	32 umol/L	5-21 umol/L
D-dimer	1.47 ugFEU/mL	0-0.5 ugFEU/mL

Because of his immunosuppression, he was urgently discussed with the microbiology consultant and it was advised to start him on IV Co-amoxiclav and continue with Clarithromycin. The microbiology team suggested further tests which are summarised in Table 2 (below) which included: Beta 2 Glucan to rule out a fungal infection (it was positive and raised at 21.23 pg/mL), Biofire (which detected human metapneumovirus), cytomegalovirus (CMV) IgM and IgG which were negative, human immunodeficiency virus (HIV) which was negative, Epstein-Barr Virus (EBV) which was detected via polymerase chain reaction (1,240 EBV DNA copies/ml). Blood and urine cultures were also sent and later came back as negative.

**Table 2:** Microbiology test results CMV: Cytomegalovirus; HIV: Human Immunodeficiency Virus; EBV: Epstein-Barr virus.

Microbiology Tests	Results	Reference value
Beta 2 Glucan	21.23 pg/mL	=>7 pg/mL POSITIVE
Biofire	Human metapneumovirus detected	N/A
CMV IgM	Negative	N/A
CMV IgG	Negative	N/A
HIV 1 and 2 Ag/Ab (EIA)	Negative	N/A
EBV	1,240 EBV DNA copies/ml	N/A
Blood cultures	Negative	N/A
Urine culture	Negative	N/A

He was also urgently discussed with the Renal team who advised to continue with his regular medications including Mycophenolate, Prednisolone and Tacrolimus. They also advised if he develops hypotension, then we can consider doubling prednisolone to 10mg/day. The medical team also decided to arrange a CTPA (CT pulmonary angiogram) and CT abdomen pelvis as urgent and the Renal team was happy for iodine contrast to be used for these scans.

CT scans showed, “Transplanted kidney in the right iliac fossa, native kidneys are relatively atrophic. Posteroinferiorly in the right thorax is a multi-septated/loculated cystic mass measuring up to 15 x 8.5 x 13 cm. Some locules contain air-fluid levels. A large vessel arising from the distal thoracic aorta courses through it, giving off multiple branches, some of which track around the periphery of the lesion. The differential includes an infected thoracic cyst. A cardiothoracic discussion is advised.” Fluid quantification via ultrasound chest of the multi-septated/loculated cystic mass was not performed.

He was discussed with the Respiratory team who believed that it was an infected cyst that will need a prolonged course of antibiotics and he was also discussed in Lung MDT, which suggested a prolonged course of antibiotics with interval imaging.

His CT scans were discussed with a cardiothoracic radiologist at a tertiary centre who suggested that the mass was likely an infected intralobar pulmonary sequestration, which was well vascularized by an anomalous bronchial artery coming directly from the descending thoracic aorta. A delayed contrast phase CT chest after five days of antibiotics was advised. It was also advised that a chest drain was absolutely contraindicated due to the risk of severe bleeding.

**Figure 2:**
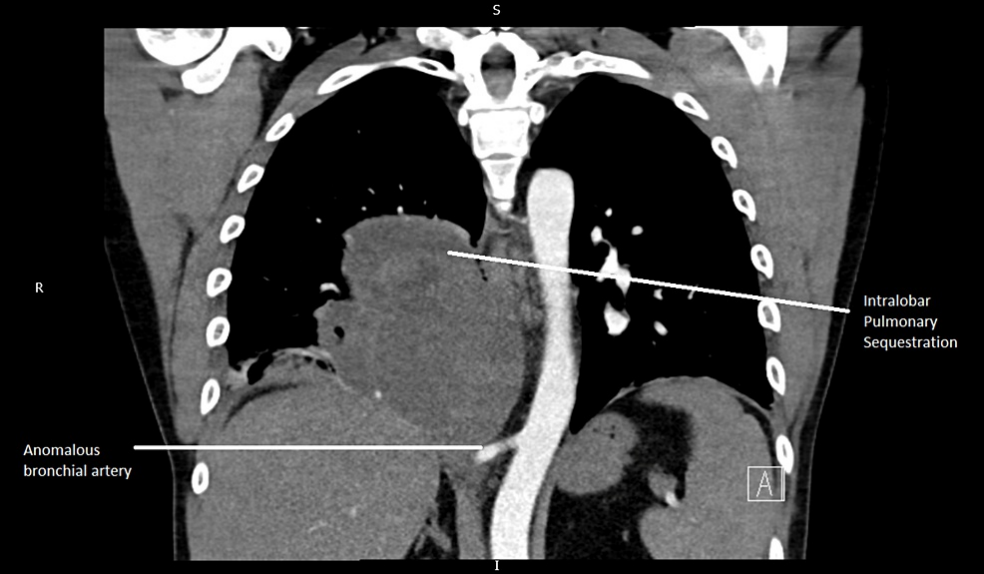
CTPA coronal section (image from the admission scan showing IPS with anomalous bronchial artery) CTPA: CT Pulmonary Angiogram; IPS: Intralobar Pulmonary Sequestration.

CTPA coronal section (image from the admission scan) is shown below (Figure 2).

**Figure 3:**
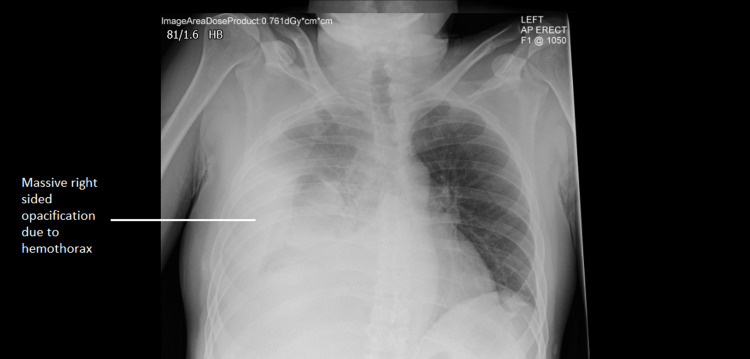
Chest X-ray showing massive right-sided opacification due to hemothorax

Eight days after admission, the patient developed acute onset of sharp, severe (8/10 in severity) back pain, radiating to the right shoulder blade and right upper quadrant of the abdomen. He also became suddenly hypotensive (with a drop of systolic BP to 91 mmHg). An urgent chest X-ray showed a massive right-sided opacification in the right hemithorax (see Figure 3).

His blood showed an acute hemoglobin drop from 147g/L to 117g/L. He was given IV fluids and an urgent CT chest was arranged. The CT chest showed an acute bleed into the right sequestrated lung and pleural space, with the likely culprit artery being the anomalous bronchial artery arising from the aorta. Although no active bleeding was present on the scan, there was a mass effect with the displacement of the aorta and the esophagus.

**Figure 4:**
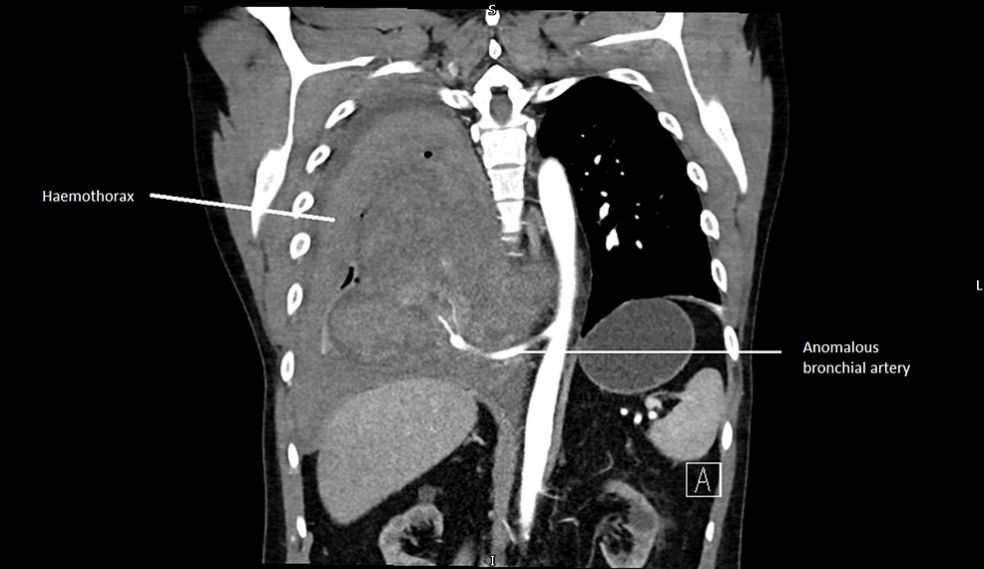
CT chest coronal image, scan done after eight days of admission showing right-sided haemothorax from anomalous bronchial artery bleed

CT chest coronal image (scan done after eight days of admission) showing right-sided haemothorax is shown below (Figure 4).

He was shifted to HDU (High Dependency Unit) and was then transferred urgently to a Cardiothoracic team at a tertiary center, where he had a right-sided chest drain inserted. The anomalous bronchial artery was embolized, and he underwent a right lower lobectomy and was started on Voriconazole for Aspergillus which was found on a Bronchial Alveolar Lavage. His sputum culture showed the growth of Pseudomonas Aeruginosa, and his antibiotics were switched to Meropenem. Voriconazole was later stopped as the resected lung lobe histology did not show any definitive evidence of an Aspergillus infection. He was later discharged with a course of oral doxycycline and made an uneventful recovery.

## Discussion

Pulmonary sequestration is a rare congenital malformation. It represents 0.15-6.4% of all congenital pulmonary malformations [1]. It is characterized by non-functional, dysplastic mass of lung tissue that is not in communication with the normal tracheobronchial tree and is associated with a systemic arterial supply. The sequestrations usually receive arterial supply from the thoracic or abdominal aorta, and have variable venous drainage into either the pulmonary veins, azygous vein or portal vein [2].

Sequestrations can be sub-divided on an anatomical basis as either intra-lobar sequestration (ILS), in which the sequestration lies within the native lung’s visceral pleural lining, or extra-lobar sequestration (ELS) which has its own pleural investment. ILS is more common (75% of cases) [3] and presents in childhood or early adulthood with recurrent pneumonia, cough, fever, hemoptysis, and chest pain [1,4,5]. ELS may be symptomatic and can present as infection/respiratory distress/cyanosis/poor feeding/congestive cardiac failure in infants due to lung hypoplasia or mass effect. Both pulmonary sequestrations show a higher male:female incidence: ILS (6:1) [6] and ELS (4:1) [7]. The differences between ILS and ELS are summarised in Table 3 (see below).

**Table 3:** Differences between intralobar and extralobar pulmonary sequestrations

INTRALOBAR SEQUESTRATION	EXTRALOBAR SEQUESTRATION
Accounts for 75% of cases	Accounts for 25% of cases
Does not have its own visceral pleura	Possesses its own pleural investments
Presents in adolescence or adulthood usually with recurrent pneumonia, chest pain, hemoptysis and rarely hemothorax	Presents in infants with infection/respiratory distress/cyanosis/poor feeding/congestive cardiac failure
Commonly gets infected	Rarely gets infected as has its pleural sac
Congenital anomalies are rare	Congenital anomalies occur more frequently including congenital diaphragmatic hernia, vertebral anomalies, congenital heart disease, pulmonary hypoplasia, foetal hydrops and colonic duplication
Higher male: female incidence 6:1	Higher male: female incidence 4:1

The aberrant artery in an intralobar pulmonary sequestration usually arises from the descending thoracic aorta in 74% of all cases [1] and the venous return in 95% of all cases is via the pulmonary veins [1]. In 97% of the cases [1], the intralobar sequestration is in the lower lobes.

Microscopically, intralobar sequestrations are characterized by cystic lesions lined by cuboidal/columnar epithelium, with lymphocyte rick inflammatory tissue along with fibrosis along with lymphocyte-rich infiltrated tissue [2].

Pulmonary sequestration is a predisposing factor of hemoptysis as the pressure from the anomalous systemic artery is higher than the pressure in the surrounding pulmonary artery vasculature. This can also lead to the development of focal pulmonary hypertension with a potential for the development of high-output cardiac failure [8]. Hemothorax is a rare presentation of intralobar pulmonary sequestration [9].

The diagnosis of pulmonary sequestrations can be accomplished through CT/MR angiography. Treatment is with percutaneous endovascular embolization of the anomalous artery, and surgery including lobectomy and pneumonectomy^ ^[3,10,11].

## Conclusions

Pulmonary sequestration is an uncommon finding in adult patients, and intralobar pulmonary sequestration usually presents with recurrent pneumonia, chest pain, hemoptysis or rarely hemothorax. CT/MR angiogram is helpful in diagnosing the anomalous arterial supply to the pulmonary sequestration. Arterial embolization +/- lobectomy is the treatment of choice for intralobar pulmonary sequestration.
